# Domestic

**Published:** 1841-04-03

**Authors:** 


### DOMESTIC.

*Death of Sir Astley Cooper.*—This distinguished surgeon died on the 12th of February, in the seventy-second year of his age.

*Weekly Report of Interments in the City and County of New York, from the* 20*th to the* 27*th of March*, 1841.—Diseases: Apoplexy, 2; asphyxia, 2; bleeding from lungs, 1; casualties, 2; cancer, 2; consumption, 34; convulsions, 12; croup or hives, 4; diarrhœa, 1; dropsy, 1; dropsy in the head, 3; dropsy in the chest, 1; dysentery, 1; erysipelas, 3; fever, 3; do. scarlet, 8; do. typhoid, 2; do. puerperal, 1; do. remittent, 1; do. intermittent, 1; intemperance, 1; inflammation, 2; do. of throat, 2; do. of brain, 5; do. of liver, 1; do. of chest, 2; do. of lungs, 22; do. of bowels, 5; do. of stomach, 3; lues venera, 1; marasmus, 4; measles, 4; old age, 4; organic disease of heart, 1; palsy, 2; suicide, 1; small pox, 4; unknown, 3; varioloid, 1.

Ages—Of 1 year and under, 25; between 1 and 2, 19; 2 and 5, 23; 5 and 10, 3; 10 and 20, 8; 20 and 30, 24; 30 and 40, 17; 40 and 50, 13; 50 and 60, 6; 60 and 70, 8; 70 and 80, 4; 80 and 90, 2.

39 men—43 women—43 boys—31 girls. Total, 153.

### HEALTH OF THE CITY.

INTERMENTS *in the City and Liberties of Philadelphia, from the 20th to the 27th of March,* 1841.

| Diseases. | Adults. | Children. |
|---|---|---|
| Abscess of lungs, | 0 | 1 |
| Apoplexy, | 1 | 0 |
| Burns, | 0 | 2 |
| Croup, | 0 | 4 |
| Cong'n of brain, | 0 | 2 |
| Consumption of the lungs, | 13 | 3 |
| Convulsions, | 1 | 8 |
| Dropsy, | 1 | 1 |
| —— head, | 0 | 7 |
| —— breast, | 0 | 1 |
| Dis'se of the brain, | 2 | 0 |
| —— heart, | 3 | 0 |
| —— lungs, | 1 | 0 |
| —— spine, | 0 | 1 |
| —— throat, | 1 | 0 |
| Debility, | 0 | 4 |
| Erysipelas, | 1 | 0 |
| Enlargement of the heart, | 1 | 0 |
| Fever, typhoid, | 0 | 2 |
| —— puerperal, | 1 | 1 |
| —— scarlet, | 0 | 1 |
| Gangrenous sore throat, | 0 | 1 |
| Hæmorrhage, | 0 | 1 |
| Inflammation of the brain, | 1 | 1 |
| —— bronchi, | 0 | 4 |
| —— lungs, | 2 | 7 |
| —— stomach, | 1 | 0 |
| —— bowels, | 0 | 1 |
| —— breast, | 0 | 1 |
| —— pleura, | 1 | 0 |
| —— peritonæum, | 1 | 0 |
| *Carried forward,* | 29 | 57 |

| Diseases. | Adults. | Children. |
|---|---|---|
| *Brought forward,* | 29 | 57 |
| Inanition, | 0 | 1 |
| Morbus cœruleus, | 0 | 1 |
| Malformation of heart, | 0 | 1 |
| Measles, | 0 | 3 |
| Mortification of intestines, | 1 | 0 |
| Neglect, | 0 | 1 |
| Old age, | 1 | 0 |
| Palsy, | 3 | 0 |
| Scrofula, | 0 | 1 |
| Small pox, | 5 | 2 |
| Still-born, | 0 | 3 |
| Suicide, | 1 | 0 |
| Teething, | 1 | 0 |
| Unknown, | 1 | 0 |
| Total, 112— | 42 | 70 |

Of the above, there

| | |
|---|---|
| were under 1 year | 27 |
| From 1 to 2 | 19 |
| 2 to 5 | 14 |
| 5 to 10 | 5 |
| 10 to 15 | 2 |
| 15 to 20 | 3 |
| 20 to 30 | 13 |
| 30 to 40 | 11 |
| 40 to 50 | 7 |
| 50 to 60 | 5 |
| 60 to 70 | 4 |
| 70 to 80 | 1 |
| 80 to 90 | 1 |
| 90 to 100 | 0 |
| Total, | 112 |

In the above are included 18 people of colour, 3 interments from the alms-house, and 1 from the country.

### BIRTHS AND DEATHS IN 1840,
*In the City and Liberties of Philadelphia.*

| Months. | Births. *Males.* | *Females.* | *Total.* | Deaths. *Males.* | *Females.* | *Total.* |
|---|---|---|---|---|---|---|
| January, | 361 | 332 | 693 | 285 | 253 | 538 |
| February, | 348 | 318 | 666 | 179 | 201 | 380 |
| March, | 365 | 376 | 741 | 269 | 247 | 516 |
| April, | 318 | 307 | 625 | 206 | 170 | 376 |
| May, | 297 | 307 | 604 | 168 | 167 | 335 |
| June, | 330 | 308 | 638 | 280 | 190 | 470 |
| July, | 392 | 334 | 726 | 270 | 265 | 535 |
| August, | 376 | 329 | 705 | 296 | 241 | 537 |
| September, | 372 | 363 | 735 | 213 | 173 | 386 |
| October, | 361 | 366 | 727 | 186 | 123 | 309 |
| November, | 329 | 339 | 668 | 128 | 136 | 264 |
| December, | 374 | 331 | 705 | 166 | 137 | 303 |
| | 4223 | 4010 | 8233 | 2646 | 2303 | 4949 |

